# Gene editing of three *BnITPK* genes in tetraploid oilseed rape leads to significant reduction of phytic acid in seeds

**DOI:** 10.1111/pbi.13380

**Published:** 2020-04-13

**Authors:** Niharika Sashidhar, Hans J. Harloff, Lizel Potgieter, Christian Jung

**Affiliations:** ^1^ Plant Breeding Institute Christian‐Albrechts‐University of Kiel Kiel Germany; ^2^ Environmental Genomics Botanical Institute Christian‐Albrechts‐University of Kiel Kiel Germany; ^3^ Environmental Genomics Max‐Planck‐Institute for Evolutionary Biology Plön Germany

**Keywords:** *Brassica napus*, *BnITPK*, CRISPR‐Cas, inositol (1, 3, 4) P3 5/6 Kinase, inorganic phosphorus, *lpa* mutants, phytic acid, polyploidy, rapeseed meal

## Abstract

Commercialization of *Brassica napus.* L (oilseed rape) meal as protein diet is gaining more attention due to its well‐balanced amino acid and protein contents. Phytic acid (PA) is a major source of phosphorus in plants but is considered as anti‐nutritive for monogastric animals including humans due to its adverse effects on essential mineral absorption. The undigested PA causes eutrophication, which potentially threatens aquatic life. PA accounts to 2‐5% in mature seeds of oilseed rape and is synthesized by complex pathways involving multiple enzymes. Breeding polyploids for recessive traits is challenging as gene functions are encoded by several paralogs. Gene redundancy often requires to knock out several gene copies to study their underlying effects. Therefore, we adopted CRISPR‐Cas9 mutagenesis to knock out three functional paralogs of *BnITPK*. We obtained low PA mutants with an increase of free phosphorus in the canola grade spring cultivar Haydn. These mutants could mark an important milestone in rapeseed breeding with an increase in protein value and no adverse effects on oil contents.

## Introduction

**Figure 1:**
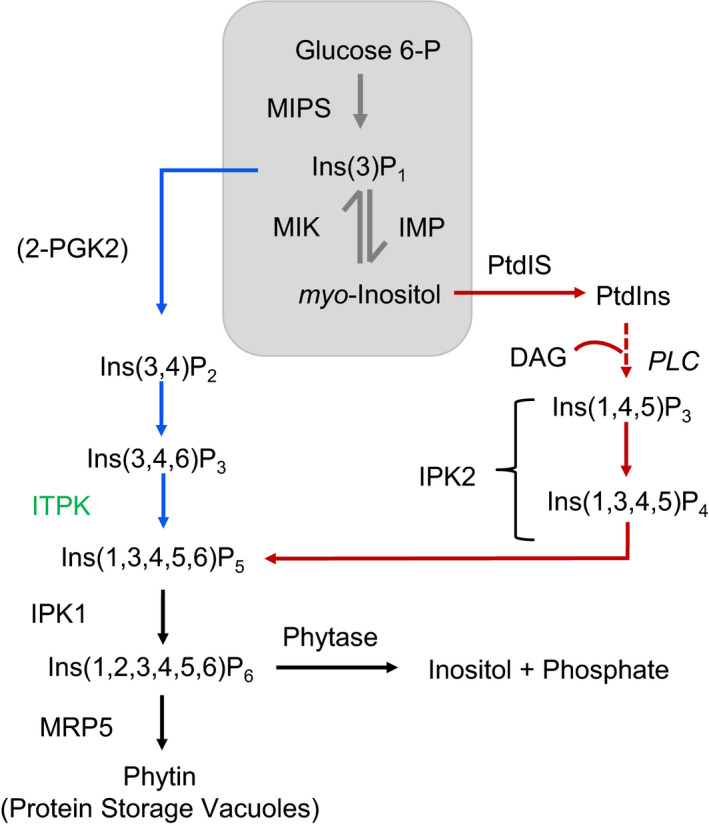
Proposed phytic acid pathway in plants (modified after Raboy et al (2009)). The grey colour box indicates the Loewus pathway, which is a housekeeping pathway for various *myo‐*inositol‐dependent pathways. Blue and red arrows indicate the lipid‐independent pathway and lipid‐dependent pathway, respectively. The targeted gene (*ITPK*) for CRISPR‐Cas9 mutagenesis is highlighted in green. MIPS: *myo*‐inositol phosphate synthase, MIK: *myo‐*inositol kinase, IMP: *myo*‐inositol monophosphatase, 2‐PGK2: 2‐phosphoglyceric acid kinase, ITPK: inositol tetrakisphosphate kinase, IPK1: inositol pentakisphosphate 2‐kinase, IPK2: inositol multiphosphate kinase, MRP5: multidrug resistance protein, DAG: diacyl glycerol, PLC: phospholipase C, PtdIS: phosphatidyl inositol synthase.

Seeds are major reserves for nutritional elements. Inositol hexakisphosphate, also known as phytic acid (PA), contributes to 65‐90% of total phosphorus in seeds across a wide range of plant species from cereals to oilseeds (Raboy, 2000). PA is a negatively charged molecule and chelates essential minerals, thereby leading to the so‐called hidden hunger (Gibson *et al.*, 2018; Perera *et al.*, 2018). Furthermore, due to lack of phytases, monogastric animals including humans cannot digest PA and the undigested PA is causing eutrophication (Raboy *et al.*, 2000; Shi *et al.*, 2007). In plants, PA is synthesized in two different ways, via a lipid‐dependent and a lipid‐independent pathway (Raboy, 2003). The PA pathway is initiated by oxidative cyclization of glucose 6‐phosphate to *myo*‐inositol monophosphate via *myo*‐inositol phosphate synthase. Subsequently, *myo*‐inositol monophosphate is converted to free *myo*‐inositol via de‐phosphorylation by *myo*‐inositol monophosphatase. Concurrently, a reverse reaction occurs by *myo*‐inositol kinase, where the free *myo*‐inositol is phosphorylated again to *myo*‐inositol monophosphate. These reactions are considered to be the housekeeping pathway for various *myo*‐inositol‐dependent pathways and commonly known as Loewus pathway (Donahue *et al.*, 2010; Loewus and Murthy, 2000). Furthermore, interaction of *myo*‐inositol with phosphatidyl lipids leads to the lipid‐dependent, whereas subsequent phosphorylation by respective kinases leads to the lipid‐independent pathway (Figure 1) (Raboy, 2009). Although both pathways contribute to PA biosynthesis, the lipid‐independent pathway is considered as the predominant pathway for PA accumulation in seeds (Raboy, 2000). Finally, the synthesized PA is transported by a multidrug‐resistant protein into vacuoles where it is stored as calcium, magnesium and potassium salts referred to as phytins (Figure 1) (Otegui *et al.*, 2002). Upon seed germination, endogenous phytases are activated and PA is hydrolysed to free inorganic phosphate (P_i_) and *myo*‐inositol (Yao *et al.*, 2012). The released P_i_ and partially phosphorylated inositols are utilized in various physiological processes during plant development including DNA repair, mRNA export, cellular signalling, and biotic and abiotic stress tolerance (Dieck *et al.*, 2012; Sparvoli and Cominelli, 2015). In oilseed rape, PA contents vary from 2 to 5%. This includes quality type (‘canola’) varieties with reduced erucic acid and glucosinolate contents (Tan *et al.*, 2011; Thompson, 1990; Uppström and Svensson 1980; Zhao *et al.*, 2008). So far, approaches to lower the PA content in extracted rapeseed meal focused either on adding phytases to the meal or to remove PA by technical processes (Adem *et al.*, 2014; El‐Batal and Abdel Karem 2001). In spite of these strategies to reduce PA, there are still several obstacles for an efficient purification of the meal (Dersjant‐Li *et al.*, 2015). In this study, we aimed to knock out the key enzyme ITPK (inositol tetrakisphosphate kinase), which is catalysing the penultimate step for the synthesis of PA in plants (Raboy, 2009). ITPK is a highly conserved enzyme across various species from plants to humans and has been shown to have diverse functions (Jiang *et al.*, 2019). It belongs to the super family of ATP‐grasp fold proteins and is involved in photomorphogenesis by interacting via the COP9 signalosome, in stress responses, in signal transduction and in seed coat development (Du *et al.*, 2011; Qin *et al.*, 2005; Sweetman *et al.*, 2007; Tang *et al.*, 2013). In our previous study, we have identified EMS (ethyl methanesulphonate)‐induced loss‐of‐function mutations in six genes of the PA biosynthesis pathway (Sashidhar *et al.*, 2019). However, due to gene redundancy in polyploid rapeseed noticeable phenotypic effects could only be observed in plants carrying multiple mutations which required time‐consuming crossing experiments. Thereby, functional analysis of traits that are encoded by several paralogs needs a fast forward approach for simultaneous knockouts. In this study, we aimed to knock out multiple copies of the key enzyme ITPK (inositol tetrakisphosphate kinase), which is catalysing the synthesis of inositol pentakisphosphate by implementing CRISPR‐Cas9‐mediated mutagenesis (Figure 1) (Zhang *et al.*, 2012), to obtain *lpa* mutants with no pleiotropic effects. Therefore, our study offers new perspectives for breeding low PA rapeseed.

## Results

### Identification of* ITPK* genes and selection of paralogs for mutagenesis

**Figure 2:**
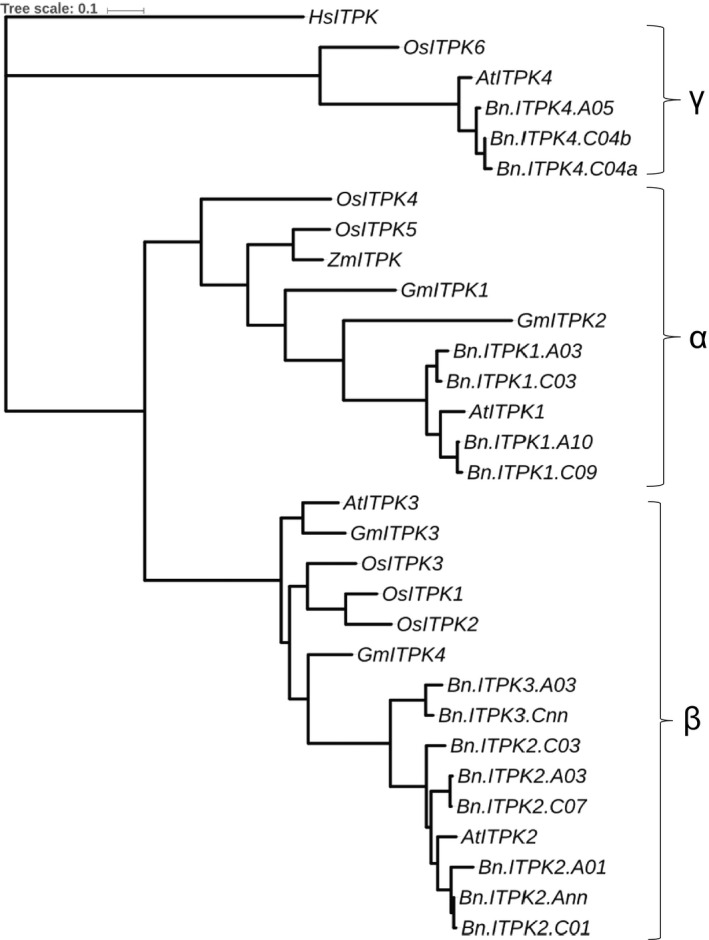
Phylogenetic tree of *ITPK* genes in different plant families. The tree was constructed using the maximum likelihood method with nucleotide substitution model as general time reversible. Bootstrapping using 500 replicates. *At* – *A. thaliana*, *Bn* – *B. napus*, *Os* – *Oryza sativa*, *Gm* – *Glycine max*, *Zm* – *Zea mays*, *Hs* – *Homo sapiens*, *Eh – Entamoeba histolytica*. Accession numbers of rice ITP5/6Ks are as follows: *OsITPK5/6‐1* (AK106544), *OsITPK5/6‐2* (AK100971), *OsITPK5/6‐3* (AK067068), *OsITPK5/6‐4* (AK071209), *OsITPK5/6‐5* (AK059148) and *OsITPK5/6‐6* (AK102571) (Kim and Tai, 2014).Arabidopsis and *B. napus* genes are mentioned in the Table 1. *Eh*: AF118848, *Hs*: NP055031, *Gm*: type 1 – EU033958, type 2 – EU033959, type 3 – EU03396h, type 4 – EU033961, *Zm*: AY172635 (Stiles *et al.*, 2008). The groups of ITPKs are shown as α, β and γ.

So far, no oilseed rape orthologs of *Arabidopsis thaliana ITPK* genes have been identified. We have used four known *AtITPK* sequences for a BLAST query in the *B. napus* database (http://www.genoscope.cns.fr/brassicanapus/) which resulted in 15 paralogs (Table 1). The gene families in *B. napus* were referred to as *BnITPK1* to *BnITPK4,* and the homology between members of the same gene family ranged between 89 and 99%. *BnITPK1* shared approx. 80% similarity with *BnITPK2* and *BnITPK3*, but less than 20% similarity with *BnITPK4* (Table 1). In order to find suitable target genes for a CRISPR‐Cas9 knockout experiments, a phylogenetic tree was constructed between *BnITPK*s and *ITPK* orthologs from other plants. The analysis resulted in three distinct clustered α, β and γ groups (Figure 2). Typically, *ITPK* orthologs which had been successfully targeted for mutations in previous studies belonged to the α and γ groups (Sparvoli and Cominelli, 2015). We expected that knocking out the orthologs in rapeseed would give comparable results and therefore focused on the α and γ group genes *BnITPK1* and *BnITPK4* (Figure 2). The *BnITPK1* and *BnITPK4* genes could be amplified by PCR in the cv. Haydn, with the exception of *Bn.ITPK4.C04a* (Table S1). However, several primer combinations failed to amplify the putative *Bn.ITPK4.C04a* ortholog from five different rapeseed genotypes (cv. Haydn, cv. Express 617, cv. Mozart, cv. Tapidor and RS 306) which was therefore excluded from further studies. For CRISPR‐Cas9 knockout studies, we choose to knock out paralogs of *BnITPK1* and *BnITPK4* gene families in a single plant.

**Table 1:** Features of *BnITPK* genes used in this study

Arabidopsis gene	*B. napus* sequence annotation	*B. napus* paralogs	Genomic sequence length (bp)	Exon/intron structure		Coding region (bp)	Protein size (aa)	Genome sequence identity with *AtITPK* (%)	Amino acid sequence identity with *AtITPK* (%)
				Exons	Introns				
*AtITPK1*	*BnaA10g17710D*	*Bn.ITPK1.A10*	960	1	0	960	320	86.7	89.3
	*BnaC09g41080D*	*Bn.ITPK1.C09*	960	1	0	960	320	86.1	89.3
	*BnaC03g07940D*	*Bn.ITPK1.C03*	975	1	0	975	325	84.8	88.4
	*BnaA03g06170D*	*Bn.ITPK1.A03*	981	1	0	981	327	84.9	88.4
*AtITPK2*	*BnaAnng34680D*	*Bn.ITPK2.Ann*	2454	9	8	1164	388	89.2	84.5
	*BnaC01g04480D*	*Bn.ITPK2.C01*	2456	9	8	1155	385	89.3	85.4
	*BnaA03g50630D*	*Bn.ITPK2.A03*	2316	9	8	1158	386	88.5	84.9
	*BnaC07g44490D*	*Bn.ITPK2.C07*	2391	9	8	1185	395	88.5	82.1
	*BnaC03g66400D*	*Bn.ITPK2.C03*	2539	10	9	1041	347	89.7	82.3
	*BnaA01g03220D*	*Bn.ITPK2.A01*	969	5	4	654	218	88.8	83.1
*AtITPK3*	*BnaCnng47190D*	*Bn.ITPK3.Cnn*	1964	9	8	1077	359	88.8	80.3
	*BnaA03g38640D*	*Bn.ITPK3.A03*	1451	8	7	930	310	82.6	80.5
*AtITPK4*	*BnaA05g03660D*	*Bn.ITPK4.A05*	2634	12	11	1467	489	88	86.9
	*BnaC04g03240D*	*Bn.ITPK4.C04a*	2455	12	11	1374	458	88.7	86.7
	*BnaC04g03250D*	*Bn.ITPK4.C04b*	2751	12	11	1452	484	89	86.7

### CRISPR‐Cas9 mutagenesis of *BnITPK* genes

**Figure 3:**
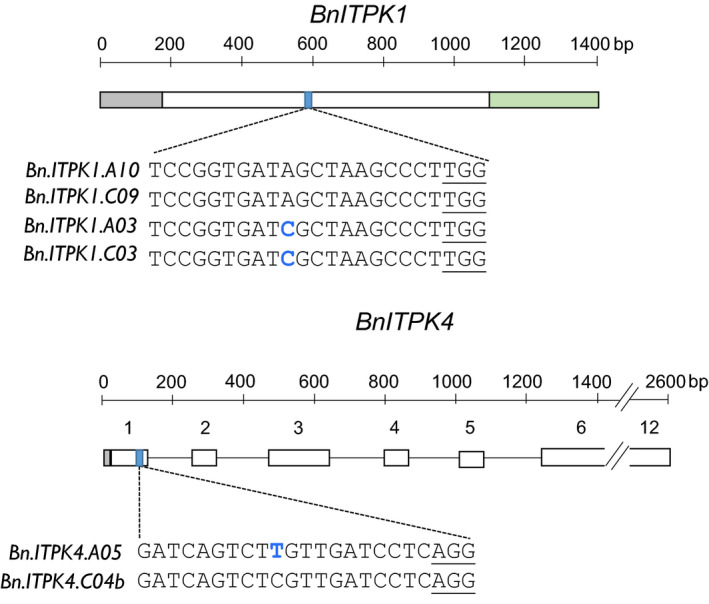
Gene structure and CRISPR‐Cas9 target sites in six *BnITPK* genes. Transcribed sequences of *BnITPK1* and *BnITPK4* gene families. Grey and green boxes indicate the 5’ UTR and 3’ UTR, respectively. Open boxes indicate exons, and the black lines indicate the introns. Blue boxes indicate target sites. The *Bn.ITPK1.A10* sequence represents target site 1, whereas the *Bn.ITPK4.C04b* sequence represents target site 2 (Express 617). SNPs are indicated by blue letters. The *Streptococcus pyogenes* PAM sequence is underlined.

Due to different gene structures between *BnITPK1* and *BnITPK4*, two different target sites, referred to as target sites 1 and 2, respectively, were chosen (Figure 3). Within each family, the target sequences differed by one nucleotide between the respective members of the family (position 10 upstream of the PAM sequence) (Figure 3). Since the BLAST search against the rapeseed reference genome did not show any off‐targets, neither in *BnITPK2* and *BnITPK3* genes nor in any other coding sequence of the rapeseed genome, these target sites were used for subsequent gene editing. Moreover, the genes containing the SNPs were lowly expressed in developing seeds of Express 617 and leaves of a Chinese semi‐winter line compared to the other members of the respective family (Shah et al. 2018) (Figure S1), indicating that they might play a minor role in PA biosynthesis in oilseed rape*.*

**Figure 4:**
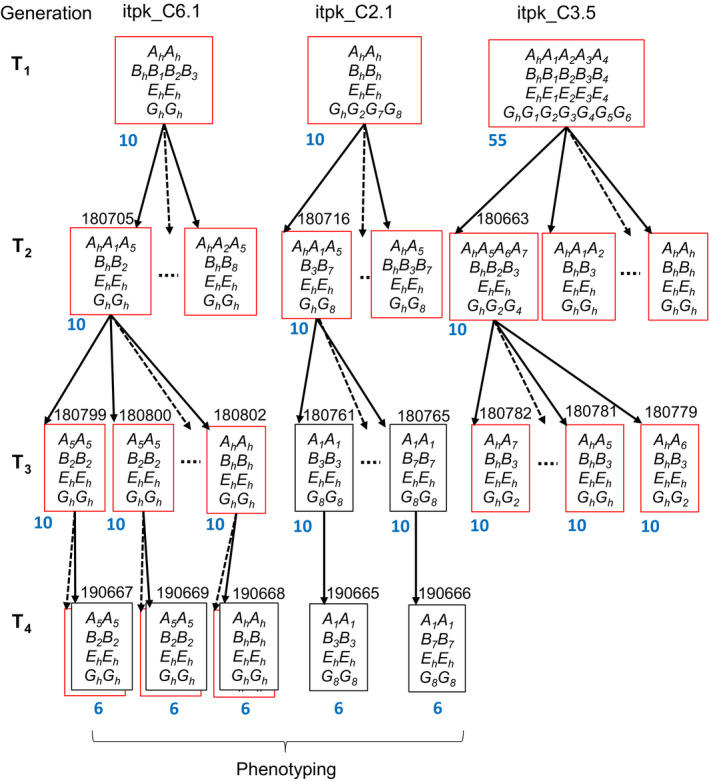
Pedigree of three *BnITPK* mutant generations derived from three different T_1_ plants. Target sites and T‐DNA insertion sites were genotyped with primer combinations given in Table S. Each box represents the mutated and non‐mutated alleles in a single plant. Red boxes indicate transgenic, and black boxes indicate non‐transgenic plants, respectively. A non‐edited Haydn allele is indicated by a suffix ‘*h*’, whereas edited alleles are numbered according to their mutation type (see Table S3). Letter codes *A*, *B*, *E* and *G* indicate alleles of *Bn.ITPK1.A10*, *Bn.ITPK1.C09*, *Bn.ITPK4.A05* and *Bn.ITPK4.C04b*, respectively. Homozygous plants used for phenotyping were obtained in the T_4_ generation. Seed codes are written on top of each genotype. Dotted arrows indicate progenies that were not further analysed in this study. The number of plants used for genotyping is given in blue colour.

Hypocotyl transformation was performed to introduce the CRISPR‐Cas9 constructs into the spring cv. Haydn. A total of 321 hypocotyls were co‐cultivated with *Agrobacterium tumefaciens* containing the Cas9‐sgRNA cassette with both target sites. From the treated hypocotyls, 23 shoots from 10 independent transgenic events (termed T_1_ generation) were regenerated to whole plants (itpk_C1 to itpk_C10) on BASTA selection media (Table S2). All T_1_ plants were tested positive for the presence of the transgene using primers flanking the *Cas9* gene (Table S1), which is corresponding to a transformation efficiency of 3.1%. PCR amplicons from leaf DNA of each transformant were sequenced to search for mutations within the targeted regions. All T_1_ plants showed gene editing in up to four genes. Three mutants were selected for further studies (itpk_C2.1, itpk_C3.5 and itpk_C6.1). Sanger sequencing revealed an overlay of more than two different sequences, suggesting that these were chimeric plants and that some mutations might have occurred somatically. Therefore, amplicons containing the target regions were cloned into plasmids and were subsequently sequenced. We observed the presence of three to eight different mutant alleles (single letter code *A, B, E, G*) for each gene along with non‐edited alleles for each of the genes targeted thus confirming the chimeric nature of the T_1_ plants (Figure 4).

### Generating homozygous double and triple mutants

We expected that chimeric T_1_ plants give rise to complex segregation patterns in the T_2_ generation and that homozygous mutants can only be selected from later generations. Of each T_2_ population, 10 (itpk_C2.1 and itpk_C6.1*)* and 55 plants (itpk_C3.5), respectively, were genotyped with locus‐specific primers (Figure 4)*.* All T_1_ mutant alleles were found in the T_2_ generations except in the itpk_C3.5 offspring where not any of the *Bn.ITPK4.A05* mutations (*E_1_*, *E_2_*, *E_3_* and *E_4_* alleles) was inherited to the next generation. We reason that these mutations were absent from the germ‐line; thus, they were not transmitted to the next generation. This was also consistent with the T_1_ plasmid sequencing results where the ratio of edited to non‐edited alleles was considerably low. Interestingly, new mutant alleles appeared in two T_2_ populations (offspring of itpk_C2.1 and itpk_C6.1) which had not been detected in their T_1_ parents (Figure 4). Furthermore, complex editing patterns were found even in T_2_ plants illustrating that Cas9 activity giving rise to *de novo* mutations in this generation. However, no editing was observed in *Bn.ITPK1.A03* and *Bn.ITPK1.C03* (*C_h_* and *D_h_*). The presence of a transgene in all analysed T_2_ plants suggested that more than one copy of the transgene was inserted which explains that Cas9 was still active in all T_2_ plants analysed. Since the mutant alleles were not fixed in T_2_ plants, three genotypes from each family (seed codes: 180716,180663 and 180705) were randomly chosen and selfed to get homozygous non‐transgenic mutants in the T_3_ generation. Firstly, the segregation ratios for the transgene loci were determined. T_3_ offspring of itpk_C2.1, itpk_C3.5 and itpk_C6.1 showed a 3:1 segregation (transgenic: non‐transgenic) indicating a single insertion event (Table S4). Secondly, homozygous double mutants (*BnITPK1*) and homozygous triple mutants (*BnITPK1* and *BnITPK4*) were identified in the T_3_ generation. Heterozygous triple mutations for *BnITPK1* and *BnITPK4* genes were found in the itpk_C3.5 progenies (Figure 4, Table S3). Finally, T_4_ populations were produced where non‐transgenic homozygous plants for the *ITPK* mutations were selected for phenotypic analysis.

### Triple mutants showed reduced PA contents and increased Pi levels

**Figure 5:**
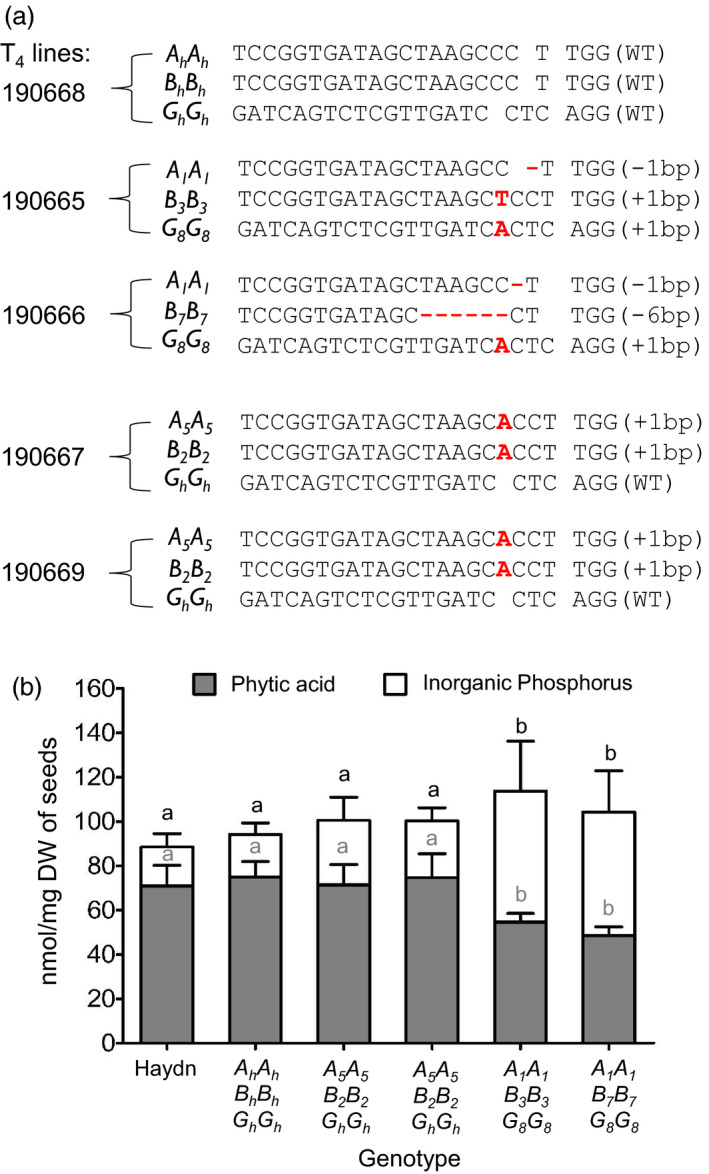
Genotypes and phenotypes of *BnITPK* mutants. (a): A non‐edited allele is indicated by a suffix ‘*h*’, while edited alleles carry numbers (see Table S3). WT stands for the Haydn allele (wild type). CRISPR‐Cas9 mutations are indicated by red letters (insertions) and by ‘‐’ (deletions). (b): Phytic acid and free inorganic P were measured in homozygous T_5_ seeds. ANOVA and post hoc test using Tukey’s multiple comparison test; *p* = 0.05 were performed for statistical significance. Similar letters indicate no significant differences.

Four T_4_ mutant lines homozygous for the mutations were chosen to examine the effect of the edited alleles. Two triple mutants (Nos. 190665 and 190666) with different *Bn.ITPK1.C09* mutant alleles and two double mutants (Nos. 190667 and 190669) with the same mutated *BnITPK1* alleles were analysed. A T_4_ line homozygous for the Haydn alleles (190668) and the donor line Haydn were used as controls (Figure 5a). Only the two triple mutants displayed significantly reduced (27.2% ‐ 35.3%) seed PA contents, while the reduction in double mutants was not significant as compared to the control plants (Figure 5b). Furthermore, no partially phosphorylated inositol phosphate intermediates could be detected. To find out whether a decreased amount of PA led to an increase of P_i_ contents in the seeds, P_i_ contents were measured in all the genotypes with the colorimetric ammonium molybdate test. As expected, the two triple mutants showed higher P_i_ contents as compared to the control. These results demonstrate that in oilseed rape, a decrease of PA is highly correlated with an increase of free phosphorus (Figure 5b). However, this increase is not molar equivalent to the reduced PA contents, which might indicate that less phosphorus is taken up by the plants.

**Figure 6:**
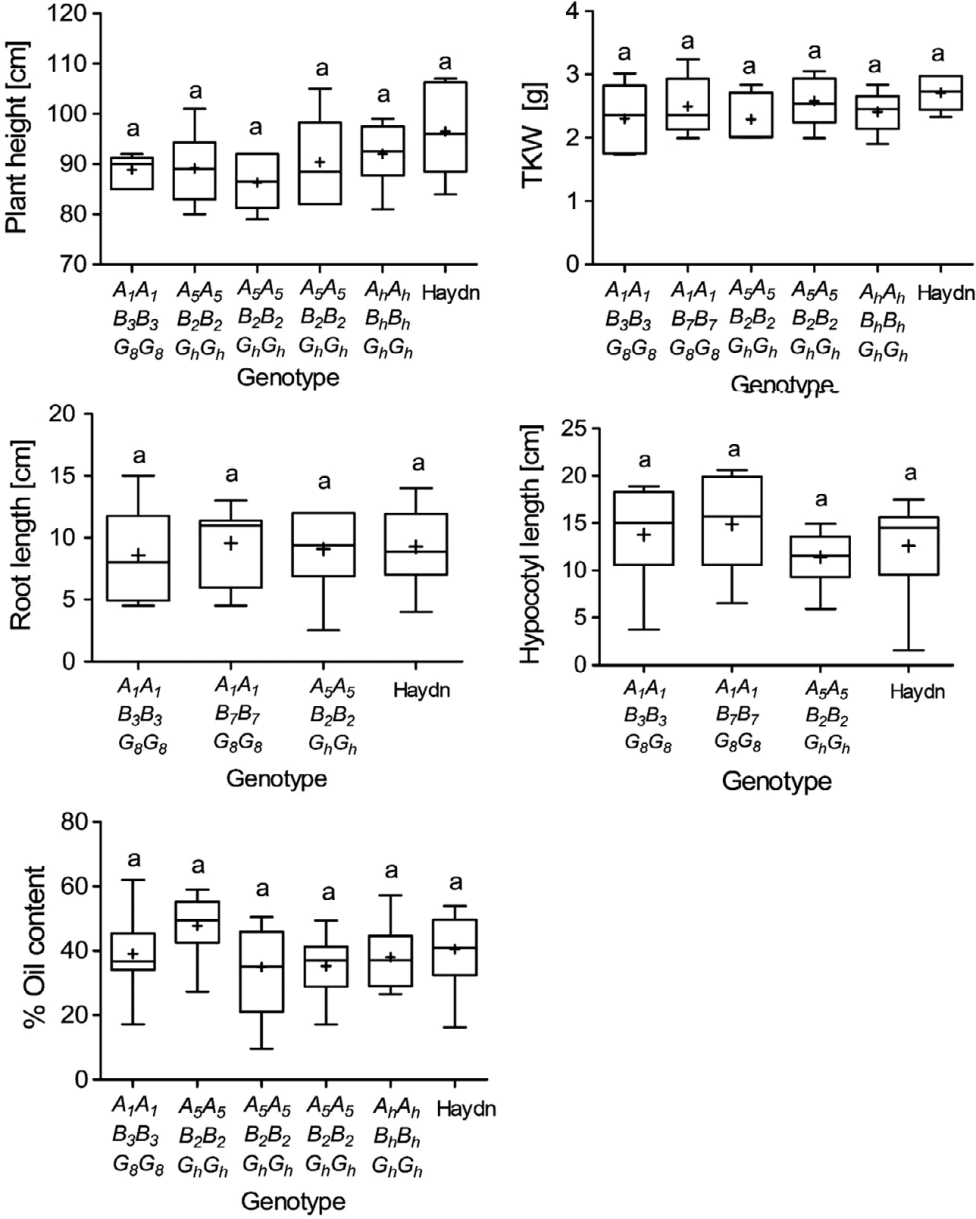
Yield‐related traits of *BnITPK* double and triple mutants. Plants were grown in the greenhouse under long day conditions (16h light/8h dark) at 22°C, and T_5_ seeds were analysed. Line 180800 is a mutant offspring carrying the Haydn alleles. Root and hypocotyl length were measured five days after germination. The oil content was measured using the n‐hexane method. Statistical significance was calculated using ANOVA followed by a post hoc test (Tukey) in R. 3.6.1. Same letters indicate no significant effect. + indicates the mean of the samples.

We questioned whether the CRISPR‐Cas9 mutations could have pleiotropic effects on yield‐related traits and seed vigour. We observed that mutant plants grew normally and did not show any obvious phenotype. Only minor differences were observed between mutant and control lines. Plant height and thousand kernel weight varied slightly between mutants. The vigour of T_5_ seedlings was evaluated five days after germination. Root length and hypocotyl length did not vary significantly as compared to the controls. The seed oil content, a major yield component of *B. napus,* was between 40 and 50%, which is within the range of modern rapeseed cultivars (Figure 6). Although the experiments were conducted under controlled conditions, the data suggest that altered PA and P_i_ contents might not have a negative impact on important agronomic traits in rapeseed.

## Discussion

This study describes low phytic acid mutants in oilseed rape by targeting genes from the PA biosynthesis pathway. We selected the *BnITPK* gene family as a suitable target for knockout analysis by CRISPR‐Cas9 in the canola grade cv. Haydn. We found a reduction of about 35% of PA with a simultaneous increase of Pi by knocking out three essential *BnITPK* genes. Interestingly, a significant reduction in PA content was only observed in triple mutants. This clearly demonstrates that knockout of multiple paralogs is necessary to observe a noticeable phenotype which can only be achieved by CRISPR‐Cas mutagenesis. Our data are in line with previous studies in wheat and potato where only multiple knockouts of different paralogs resulted in a desired phenotype (Andersson *et al.*, 2017; Wang *et al.*, 2014; Zhang *et al.*, 2019). In rapeseed, the situation is even more complicated because during evolution, the genome underwent several whole‐genome duplication and triplication events resulting in an average copy number of two to eight for each Arabidopsis ortholog (Chalhoub et al. 2014). Another important aspect of our study adds to our understanding of CRISPR‐Cas mutations during plant development and between generations. We observed mutations in T_2_ generation that had not been found in their T_1_ parents. One reason could be that only a small sector of the T_1_ plant carried the mutation, which was not detected by sequencing. However, we favour another explanation that de novo mutations appeared in the T_2_ generation. This could be due to the low activity of the Cas9 gene which is influenced by the position of the T‐DNA in the genome or by multiple T‐DNA insertions which can result in low transcriptional activities or even gene silencing (De Buck *et al.*, 2009; Tang *et al.*, 2006). It is also worth mentioning that in this study, Cas9 acted specifically and did not tolerate SNPs in the target site for inducing mutations, which is in contrast to our experiments performed on *BnALC* genes in *B. napus* (Braatz *et al.*, 2017). In conclusion, future studies should carefully examine also later generations even if T_1_ plants were lacking mutations.

Our study adds to our understanding of PA biosynthesis and storage in rapeseed. We demonstrate that ITPKs play an important role in PA metabolism and thus are a suitable target for reducing PA amounts. The *ITPK* gene was first identified in maize *lpa2* mutants (*zmitpk*) which showed a 30% reduction in PA without any effect on plant performance. Subsequently, knockout mutants with reduced PA contents in seeds have also been described in rice, Arabidopsis and maize (Raboy, 2009; Shi *et al.*, 2003). A study in rice identified two knockout mutants of *OsITPK6* showing 46% reduction of PA seed content caused by a mis‐sense mutation and 68% caused by a splice site mutation without any adverse effects on seedling performance (Kim and Tai, 2011). Another study in rice showed contradictory result. While the PA content was reduced by 32% due to the loss of two amino acids in the OsITPK6 protein, negative effects on seed set, seed weight and germination were reported (Jiang *et al.*, 2019). Mutants in Arabidopsis *atitpk1* and *atipk4* showed a 46% and 51% reduction, respectively, with no pleiotropic effects on seed germination and seedling performances (Kim and Tai, 2011). We also did not observe any negative effects on germination rate and seedling vigour which suggests that PA reduction in our mutants is below a critical threshold. We observed that the triple mutant with a 6‐bp deletion (line No. 190666) had the lowest PA content. According to protein modelling, this mutation results in the loss of two amino acids within an ATP‐binding site (see Figure S2, Figure S3) which has also been proposed for a *StITPK1* ortholog in potato (Caddick *et al.*, 2008). Although the contribution of each gene to the synthesis of PA in *B. napus* is unknown, we reason that *BnITPK* genes act in an additive manner and that an even higher reduction could be achieved by a complete knockout of all *BnITPK1* and *BnITPK4* genes. It is important to know what happens to inorganic seed Pi if it is not bound in PA. Due to the loss of PA, the triple mutants showed a threefold increase of P_i_. Similar results were obtained with a maize *lpa2* mutant where the P_i_ content was increased by threefold (Shi *et al.*, 2003). The lower increase of Pi was also shown previously as a general pattern of *itpk* mutants in rice and Arabidopsis with decreased contents of PA accompanied by a minor increase of P_i_ and lower inositol phosphates (Sparvoli and Cominelli, 2015). It is noteworthy that in our study, the increase in seed Pi was also not a molar equivalent to the reduced PA contents, which might indicate that less phosphorus is needed by the mutants.

Lowering the PA amounts in seeds is an important milestone for enhancing rapeseed meal quality for human and animal diets. Moreover, the mutants offer new perspectives for oilseed rape breeding. 1) Reduced PA content will be beneficial to combat the mineral deficiencies of the meal. 2) Recovery of rapeseed protein will be more efficient due to lower the amount of tightly bound PA. 3) Protein contents will be improved with no effect on oil contents and thus overruling their negative correlation (Jasinski *et al.*, 2018). 4) Increase of Pi contents might pave ways to reduced dependency on phosphate fertilizers, which is a non‐renewable resource (Cordell *et al.*, 2009). 5) Increased bioavailability of Pi attenuates eutrophication of waters (Conley *et al.*, 2009). *B. napus* is primarily an oil crop but can also be used as a nutritional source due to its rich protein and well‐balanced amino acid contents for human and animal diets (Campbell et al. 2016; Wanasundara *et al.*, 2016). There is increasing effort to improve the nutritional value of *B*. *napus* meal in order to overcome the dependence on the expensive soybean imports in regions where soybean cannot be grown (Jasinski *et al.*, 2018). So far, rapeseed meal is used for aquatic and poultry industries as a feed, but it can also be used as a valuable protein source for humans and non‐ruminants (Gacek *et al.*, 2018). However, the presence of various anti‐nutritive compounds like phytic acid, sinapine, fibre and glucosinolates impedes its commercialization as a major protein source. While rapeseed cultivars and lines exist which are low in glucosinolates, sinapic acid and erucic acid, no reduced PA lines are available. Furthermore, the protein extraction from seeds is impaired by the presence of tightly bound phytins and tannins (Wanasundara *et al.*, 2016), which reduces the digestion by enzymes like trypsin and pepsin but also inhibits α‐amylases at physiological pH in monogastric animals (Reddy *et al.*, 1996). Therefore, the established mutants pave the way to breed rapeseed with improved meal quality and reduced phosphorous demand.

## Experimental procedures

### Plant material and growth conditions

The German spring cultivar Haydn (double low ‘00’ or ‘canola’ quality) was used for transformation experiments. Greenhouse experiments were performed under long day conditions (16h light and 8h dark) at 22°C. Plants were grown in 11x 11cm pots, and they were not fertilized to avoid phosphorous contamination. Upon flowering, the inflorescences were bagged, and dry mature seeds were harvested. For phenotyping experiments, five biological replicates for each genotype were grown. Plants were randomized every week, and plant height (length between base of the stem to the tip of the inflorescence) and thousand kernel weight (TKW) were measured at the time of harvesting. Seed oil content was measured using the n‐hexane method. To measure the seedling’s vigour, 20 seeds of each genotype were sown on ½ Murashige–Skoog** (**MS) medium and germinated in the dark. Five days after germination, the root and hypocotyl lengths were measured.

### Phylogenetic analysis

Published *ITPK* sequences were retrieved from respective crop’s databases. Sequences from rice were obtained from http://rice.plantbiology.msu.edu/cgi‐bin/gbrowse/rice/#search , Arabidopsis from TAIR (https://www.arabidopsis.org/servlets/Search?action=new_search&type=gene), and soybean, maize and human sequences were retrieved from NCBI (https://www.ncbi.nlm.nih.gov/). The amino acid sequences of the ITPK proteins were aligned using MAFFT v7.313 with the G‐INS‐i strategy (Katoh and Standley, 2013). The alignment was manually curated. The substitution model for the maximum likelihood analysis of the curated alignment was determined by ProtTest v3.4.2 (Abascal *et al.*, 2005). The maximum likelihood phylogenetic analysis was conducted with MEGA X (Kumar *et al.*, 2018). A JTT model was applied, and 500 bootstrap replicates were performed. The resulting phylogenetic tree was visualized with iTOL (Letunic and Bork, 2007; Letunic and Bork, 2019).

### Target site design and plasmid vector construction

The vectors pChimera and pCas9_TPC were obtained from Prof. Holger Puchta (Karlsruhe institute of technology, Germany). pChimera contains the sgRNA sequence driven by an *Arabidopsis thaliana* U6‐26 promoter and pCas9_TPC vector has the Cas9 gene from *Streptococcus pyogenes* driven by an Ubi4‐2 promoter from parsley. These vectors have ampicillin and spectinomycin resistance genes, respectively, as selective markers for bacteria and BASTA resistance to select transgenic shoots (Fauser *et al.*, 2014).

The *BnITPK* CRISPR‐Cas9 cassette was constructed according to published protocols (Fauser *et al.*, 2014) with minor modifications. sgRNAs targets were cloned into the pCas9‐TPC vector by using *Avr*II and *Bcu*I restriction enzymes, as these are isocaudomeres. The final sgRNA‐target vector was transformed into the *Agrobacterium tumefaciens* strain GV3101 pMP90RK, which was then used for rapeseed hypocotyl transformation.

We used four Arabidopsis sequences encoding *ITPK* genes to BLAST against the rapeseed reference genome (http://www.genoscope.cns.fr/brassicanapus/) (Kim and Tai, 2011)*.* Retrieved sequences were annotated according to the Genoscope browser using the CLC Main Work Bench 7.9.1 (CLC, Aarhus, Denmark) software. For the target design, all paralogs of each *ITPK* gene family were aligned and regions of 20bp length and a GC content> 40% were chosen which were next to a PAM sequence (NGG). Conserved regions with a maximum of one SNP upstream of 10^th^ position from the PAM site were considered as suitable targets because they can be tolerated by Cas9 (Braatz *et al.*, 2017). All target sites chosen were blasted against the *B. napus* genome database using CLC Main Work Bench 7.0.3 for verification of any off‐targets. Sequences lacking BLAST hits were selected for further analysis. Oligonucleotides were ordered from Eurofins Genomics Company (Ebersberg, Germany) and purified by a high purity salt free (HPSF) method with a purity of> 70 %. (http://www.eurofins.de/de‐de.aspx).

### Rapeseed hypocotyl transformation

We used a protocol kindly provided by Dr. José Orsini (Saaten‐Union Biotec GmbH, Germany) with minor adaptations from a published protocol (Zarhloul et al. 1999). Seeds from cultivar Haydn were surface sterilized with 70% EtOH for two minutes followed by 3% sodium hypochlorite and 0.01% Tween 20 for 10 min. Seeds were washed thoroughly with autoclaved water and sown on MS medium. Five‐day‐old hypocotyls were used for transformation. The hypocotyls were cut into 1cm explants and incubated for 45 to 60 min with an *A. tumefaciens* overnight culture adjusted to OD_600_ 1.2. The hypocotyls were co‐cultivated for two days and transferred to shoot‐inducing medium containing 500 mg L^‐1^ ticarcillin disodium/clavulanate potassium (Duchefa‐T0190.0002). After 4 weeks on shoot‐inducing medium, the regenerated shoots were transferred to shoot‐inducing medium containing 5 mg L^‐1^ BASTA (phosphinothricin) (Duchefa: P0159.0250). Surviving shoots were transferred to root‐inducing medium for regeneration of complete plants.

The transformation efficiency was calculated as (number of independent transgenic plants/ total number of explants used) * 100.

### Verification of the mutations by molecular marker analysis

Paralog‐specific primers were designed using the Darmor‐*bzh* reference sequence to amplify the individual copies according to the protocol (Table S) (Liu *et al.*, 2012). DNA was isolated from the leaf samples using the CTAB method. Sanger sequencing was performed from the paralog‐specific amplicons to verify the putative editing events. Furthermore, PCR amplicons from chimeric T_1_ plants were cloned into the pGEM‐T vector (Promega, Germany) and plasmids were Sanger‐sequenced. For verification of the underlying alleles in the chimeric plants in the T_2_ generation, we used an online software tool called Tracking of InDels by Decomposition (TIDE) (https://tide.deskgen.com/) (Brinkman *et al.*, 2014). Raw Sanger sequencing reads of individual paralogs (.abi files) were used as query, which were then compared to the provided reference sequences (non‐edited sequences). The analysis resulted in plots for each sequence of a gene showing the probable nucleotide changes caused by the NHEJ repair mechanism.

### Measuring seed phytic acid and inorganic phosphorous contents

Phytic acid and inorganic phosphorus (Pi) were extracted following the procedure of (Matthäus *et al.*, 1995) with slight modifications. Dry matured seeds of 200 mg were ground into fine powder from five biological replicates and from each sample, three technical repeats of ~ 50 mg were analysed. Defatting was done by extraction with 1 ml n‐hexane overnight at room temperature on an overhead shaker. After centrifugation (10 min at 20,800 g), the defatted seed cake was extracted with 1 ml of 0.5 M HCl by vigorous shaking on a Bioer mixing block (MB‐102) at 30°C for 4h. After centrifugation at 20,800 g, the supernatant was freeze‐dried and re‐dissolved in 1 ml double distilled water. A 0.5‐ml aliquot was applied on a 2 ml column of Dowex 1x2 (Acros Organics, A0346424), washed with 2x10 ml water and eluted with 25 ml 2 M HCl. The eluates were freeze‐dried and re‐dissolved in 1 ml double distilled water. Phosphoinositols (IP1 to IP6) were analysed by HPLC following the procedure published by (Rounds and Nielsen, 1993) with minor modifications. Fifty‐microlitre samples were injected onto a PL‐SAX 1000 HPLC column (50 x 4.6 mm, 5µ, Agilent), and IP1 to IP6 were eluted at 30°C with a flow rate of 1 ml min^‐1^. A 25‐min linear gradient from 0.01 M 1‐methylpiperazine (pH 4.0) to 0.5 M NaNO_3_ dissolved in 0.01 M 1‐methylpiperazine (pH 4.0) with a further elution for 2 min, 1 min returning to starting conditions and 10 min equilibration was used. Analytes were detected at 500 nm using a Pinnacle PCX device (LCTech, Germany) with a post‐column reagent consisting of 0.015% (w/v) FeCl_3_ x 6H_2_O and 0.15% (w/v) sulphosalicylic acid. A calibration curve was plotted using 10‐50 µg of PA sodium salt hydrate (Sigma: P8810). For determining Pi contents, 50µl of the purified column extracts were mixed with 500 µl of colouring reagent (10% w/v of ascorbic acid (Roth‐ Art.Nr.3666.1) and 5% w/v of ammonium molybdate (Roth, Art. No.3525.2), adjusted to 1.5 ml with double distilled water and incubated at 40°C for one hour. The samples were measured against a reagent blank in a spectrophotometer at 655 nm. A calibration curve 0 to 100 nmol Pi was used.

### Statistical analysis

For greenhouse experiment, five plants of each line were grown (biological replicates). Of each plant, three technical replicates were taken for PA, inorganic P and oil measurements. An ANOVA was performed using R 3.6.0 and the MulticomView package, and post hoc test was done by using Tukey’s multiple comparison test; *p* = 0.05.

## Conflict of interest

The authors have no conflict of interest in relation to this work.

## Author contributions

N.S planned, performed and analysed the experiments and wrote the article; L.P. performed the phylogenetic analysis; H.‐J.H. and C.J. designed the study and supervised the experiments; and H.‐J.H and C.J. revised the manuscript. All authors read and approved the final manuscript.

## Supporting information


**Figure S1 **Expression analysis of *BnITPK* paralogs in seeds and leaves.
**Figure S2 **Protein alignment of ITPK showing the inositol, ATP and Mg2+ binding sites.
**Figure S3** Predicted protein structure of *Bn.ITPK1.C09* in Haydn and in the mutant line 190666.
**Table S1 **Primer sequences used in this study.
**Table S2** Editing status in regenerated T_1_ plants.
**Table S3** Gene acronyms for four *BnITPK* genes used in this study.
**Table S4** Segregation analysis in T_3_ generations.
