# Supplementary material for: Gene editing of three BnITPK genes in tetraploid oilseed rape leads to significant reduction of phytic acid in seeds
Source: Plant Biotechnol J. 2020 Apr 13;18(11):2241–50. doi: 10.1111/pbi.13380 (PMC7589381; doi:10.1111/pbi.13380)
Supplement: Supplementary file 1 — Figure S1 Expression analysis of BnITPK paralogs in seeds and leaves. Figure S2 Protein alignment of ITPK showing the inositol, ATP and Mg2+ binding sites. Figure S3 Predicted protein structure of Bn.ITPK1.C09 in Haydn and in the mutant line 190666. Table S1 Primer sequences used in this study. Table S2 Editing status in regenerated T1 plants. Table S3 Gene acronyms for four BnITPK genes used in this study. Table S4 Segregation analysis in T3 generations. [file PBI-18-2241-s001.pdf]

Gene editing of three *BnITPK* genes in tetraploid oilseed rape leads to significant reduction of phytic acid in seeds

Niharika Sashidhar, Hans J Harloff, Lizel Potgieter, Christian Jung

Supplementary Figure 1: Expression analysis of *BnITPK* paralogs in seeds and leaves. Seed samples were collected from Express 617 at different time points. Normalization was done using *BnActin2* as a reference gene. Leaf data were taken from an RNAseq study Shah et al. (2018). Y-axis represents the number of reads mapped to the reference sequence Darmor-*bzh*. NV, non-vernalized; V, vernalized. The BBCH scale stands for different developmental stages of the plant (https://www.canolacouncil.org/canola-encyclopedia/crop-development/growth-stages/).

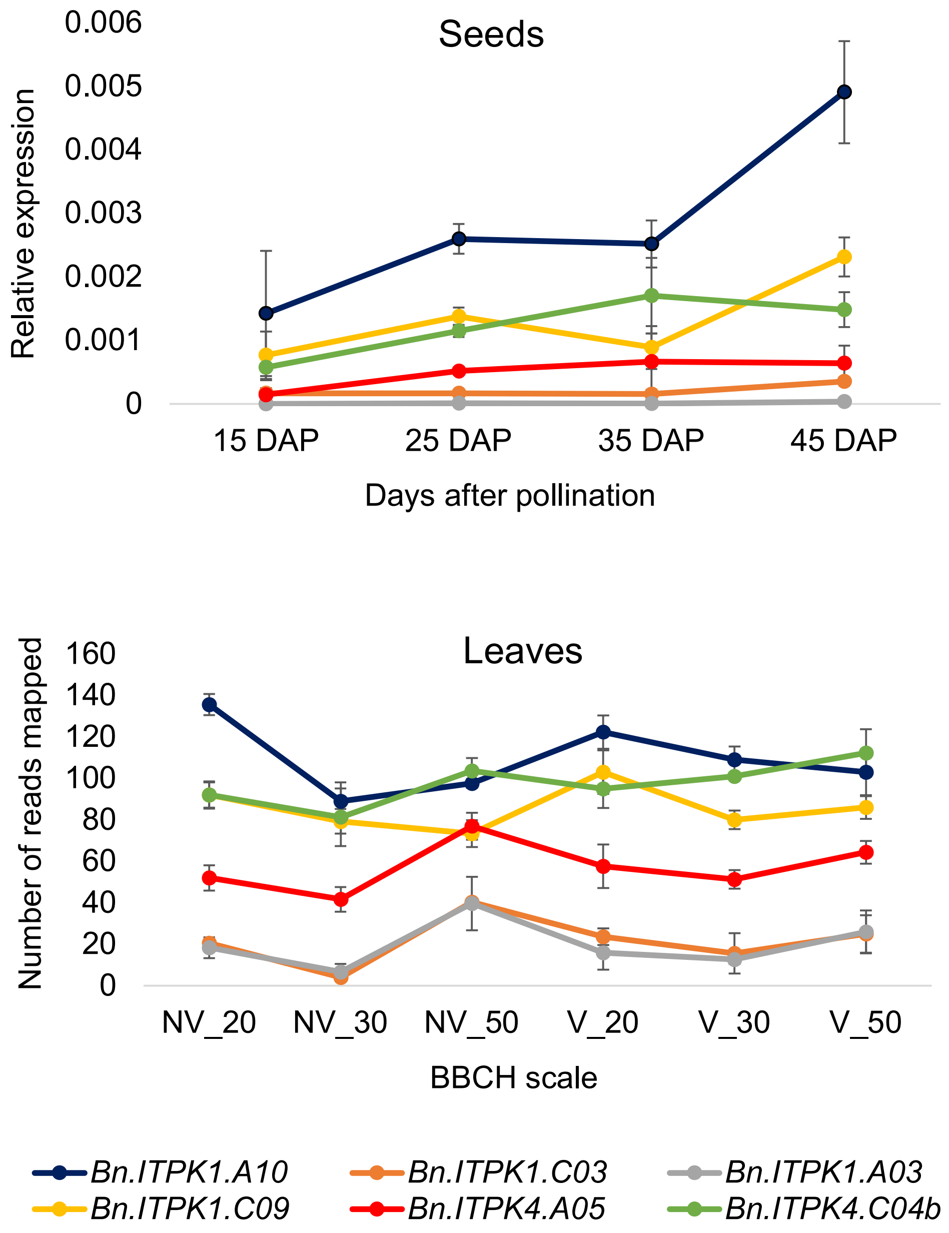

Supplementary Figure 2: Protein alignment of ITPK showing the inositol, ATP and Mg^{2+} binding sites. Inositol binding residues are shown yellow; ATP binding sites are shown in maroon and Mg^{2+} residues are shown in green. The target sites of *BnITPK1* and *BnITPK4* are shown in blue, where target site 1 contains one of the ATP binding sites (PVIA<u>K</u>P).

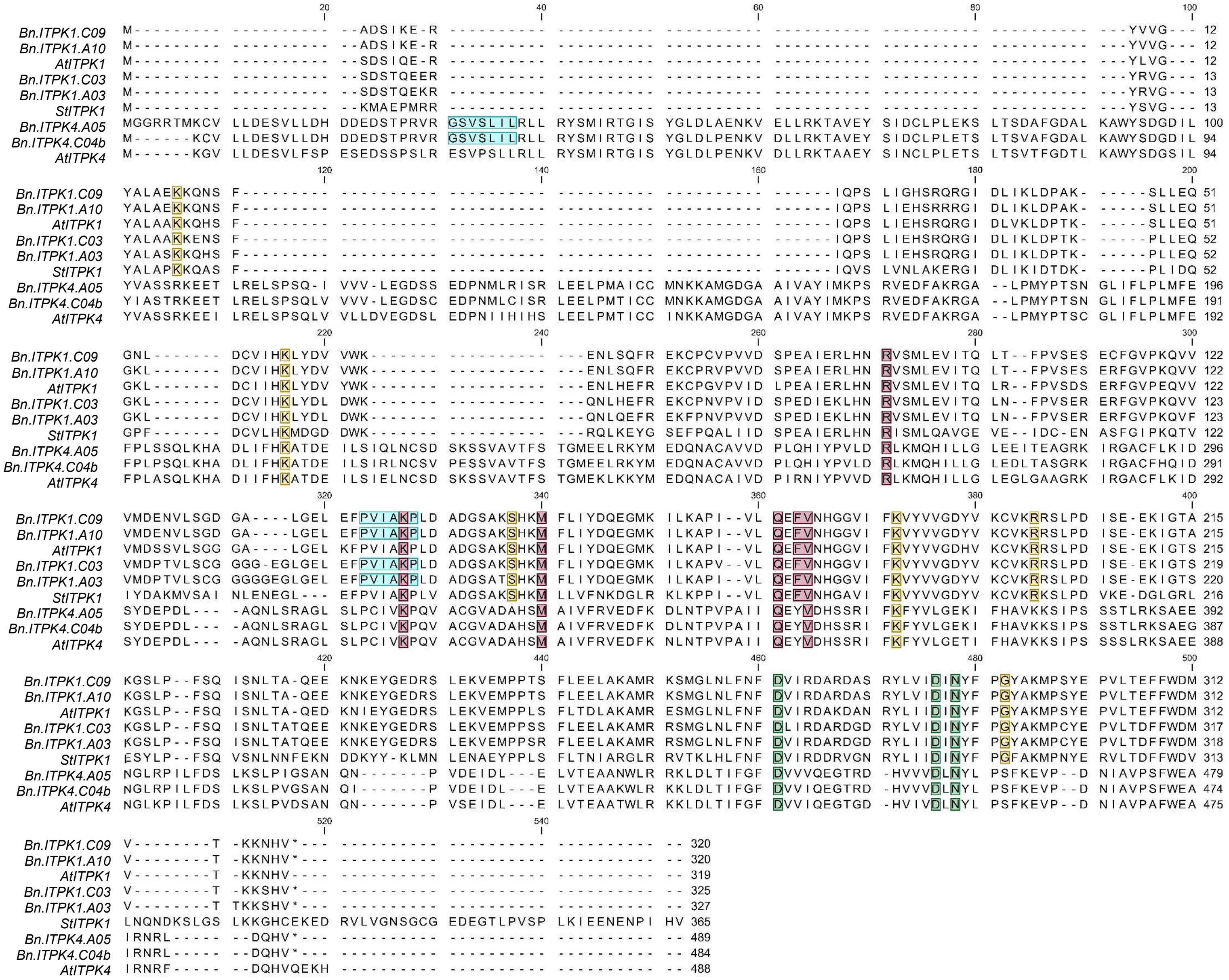

Supplementary Figure 3: Predicted protein structure of *Bn.ITPK1.C09* in Haydn and in the mutant line 190666. Protein sequences from Haydn and from the triple mutant line 190666 carrying the B_5 allele were used as query in Expasy- SWISS-Model (https://swissmodel.expasy.org/interactive). The mutant has a different protein folding structure (marked with green triangles) at one of the ATP binding sites (amino acid 'K').

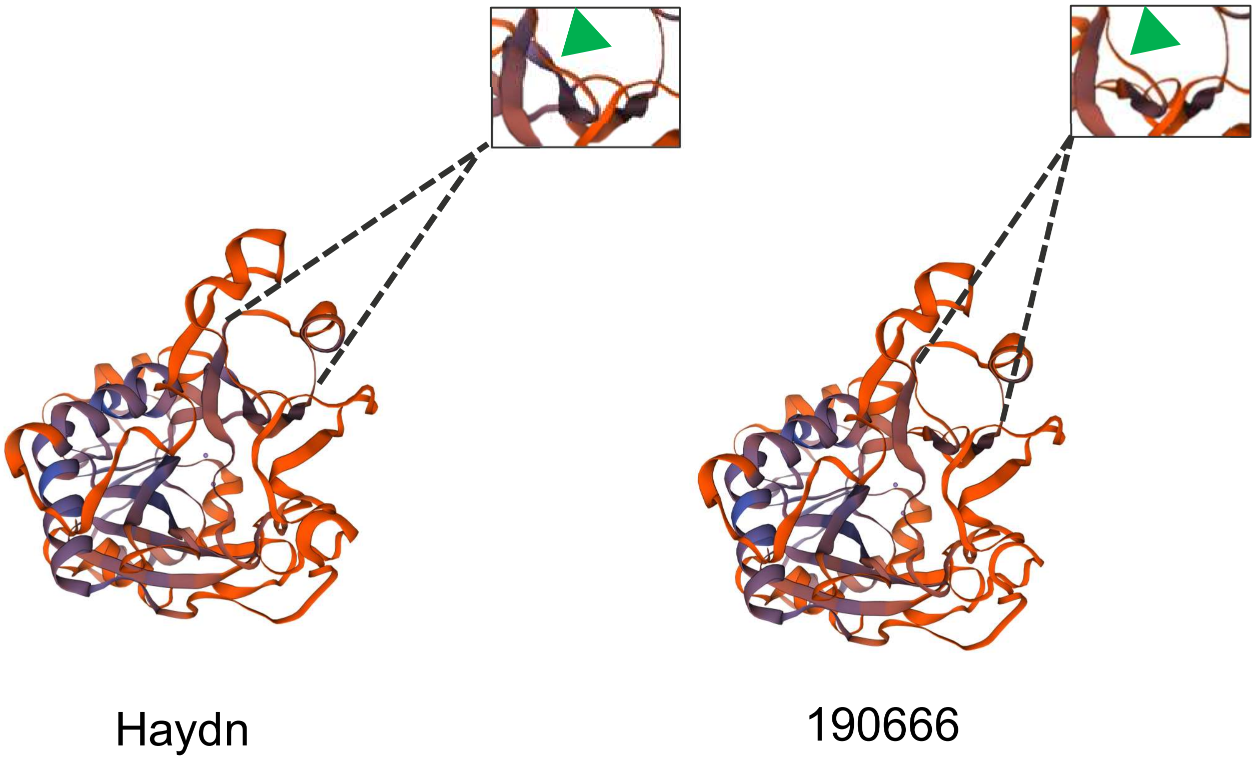

Supplementary Table 1: Primer sequences used in this study.

Gene family	Gene/target	Gene nomenclature	Primer name	Primer type	Primer sequence*	Temp (°C)	Amplicon length (bp)
BnITPK1	BnaA10g17710D	*Bn.ITPK1.A10*	NS_P138	Forward	GTTAACGTTTCCTGTATCGGAAGGT	60	259
			NS_P147	Reverse	GTAGACCTTAAAGATCACACCGTCA		
	BnaC09g41080D	*Bn.ITPK1.C09*	NS_P222	Forward	GCATGAATGTTAACCTAACTTCCTC	60	599
			NS_P274	Reverse	CGTGATTCACAAACTCCTGGAG		
	BnaC03g07940D	*Bn.ITPK1.C03*	NS_P290	Forward	GTGCCCTAACGTCCCTGCCA	63	295
			NS_P275	Reverse	CTTGAGTATCTTCATCCCTTCTCGG		
	BnaA03g06170D	*Bn.ITPK1.A03*	NS_P226	Forward	GCTCTCGCATCCAAGAAACGAC	61	753
			NS_P146	Reverse	CTTCGCCAACTCCTCCAAGTAC		
BnITPK4	BnaA05g03660D	*Bn.ITPK4.A05*	NS_P277	Forward	TCGATCACGATGATGAAGATGCA	60	428
			NS_P135	Reverse	CTTCTCCAAAGGTAAGCAATCTCTG		
	BnaC04g03250D	*Bn.ITPK4.C04b*	NS_P279	Forward	AATGTGTTTTACTCGACGAGTGTG	60	321
			NS_P278	Reverse	TGTTTTCCGGAAGATCAAGGCC		
	Chimera	-	pChimera_f	Forward	AGTGAGCGCGACGTAATACG	58	702
			pChimera_r	Reverse	GTTAATGCAGCTGGCACGAC		
	Cas9	*SpCas9*	pCas9_f	Forward	CAGTCTTTCACCTCTCTTTGG	58	1500
			pCas9_r	Reverse	CCATCTTTGGGACCACTGTC		
	Spectinomycin	-	Spec_for	Forward	TGCCGACTACCTTGGTGATC	58	910
			Spec_rev	Reverse	GTACAGTCTATGCCTCGGGC		
BnACTIN2	XM_013786210.1	-	Act1	Forward	TCTGGTGATGGTGTGTCTCA	60	141
	XM_013888645.1	-					
	XM_013893921.1	-					
	XM_013829442.1	-	Act2	Reverse	GGTGAACATGTACCCTCTCTCG		
	XM_013830703.1	-					

*Green letters indicate SNPs between the paralogs, red letters indicate the modified base to increase the primer specificity and blue letters indicate SNPs between reference sequence Darmor-*bzh* and Haydn.

Supplementary Table 2: Editing status in regenerated T_1 plants

Seed code	T_1 plants	Genotype[§]
180906	*itpk_C1.1*	$A_{ch}B_{h}C_{h}D_{h}E_{h}G_{h}$
**180624**	***itpk_C2.1***	$\boldsymbol{A_{h}B_{h}C_{h}D_{h}E_{h}G_{2,7,8}}$
180625	*itpk_C2.2*	$A_{h}B_{ch}C_{h}D_{h}E_{h}G_{ch}$
180626	*itpk_C2.3*	$A_{h}B_{h}C_{h}D_{h}E_{h}G_{h}$
180616	*itpk_C3.1*	$A_{h}B_{h}C_{h}D_{h}E_{h}G_{h}$
180617	*itpk_C3.2*	$A_{h}B_{h}C_{h}D_{h}E_{h}G_{h}$
180618	*itpk_C3.3*	$A_{h}B_{h}C_{h}D_{h}E_{h}G_{h}$
180619	*itpk_C3.4*	$A_{h}B_{h}C_{h}D_{h}E_{h}G_{h}$
**180620**	***itpk_C3.5***	$\boldsymbol{A_{1,2,3,4}B_{1,2,3,4}C_{h}D_{h}E_{1,2,3,4}G_{1,2,3,4,5,6}}$
180621	*itpk_C3.6*	$A_{ch}Bc_{h}C_{h}D_{h}E_{ch}G_{ch}$
180622	*itpk_C3.7*	$A_{ch}Bc_{h}C_{h}D_{h}E_{ch}G_{ch}$
180623	*itpk_C3.8*	$A_{ch}Bc_{h}C_{h}D_{h}E_{ch}G_{ch}$
180907	*itpk_C4.1*	$A_{ch}B_{h}C_{h}D_{h}E_{h}G_{h}$
190662	*itpk_C4.2*	$A_{ch}B_{h}C_{h}D_{h}E_{h}G_{h}$
180908	*itpk_C5.1*	$A_{ch}B_{h}C_{h}D_{h}E_{h}G_{h}$
**180627**	***itpk_C6.1***	$\boldsymbol{A_{h}B_{1,2,3}C_{h}D_{h}E_{h}G_{h}}$
180628	*itpk_C6.2*	$A_{h}B_{ch}C_{h}D_{h}E_{h}G_{h}$
180629	*itpk_C6.3*	$A_{h}B_{ch}C_{h}D_{h}E_{h}G_{h}$
180630	*itpk_C6.4*	$A_{h}B_{ch}C_{h}D_{h}E_{h}G_{h}$
180909	*itpk_C7.1*	$A_{ch}B_{h}C_{h}D_{h}E_{h}G_{h}$
190663	*itpk_C8.1*	$A_{ch}B_{h}C_{h}D_{h}E_{h}G_{h}$
190664	*itpk_C9.1*	$A_{ch}B_{h}C_{h}D_{h}E_{h}G_{h}$
190844	*itpk_C10.1*	$A_{h}B_{ch}C_{h}D_{h}E_{h}G_{h}$

[§] *ch* indicates that the plants had more than two alleles, indicating the chimeric nature of the plant. *h* indicates the donor allele from cv. Haydn. The genotypes indicated in bold were used for further propagation of T_2 generation and only these plant DNAs were cloned into plasmids for analyzing the type of mutant alleles. See Supplementary Table 3 for allele nomenclature codes.

Supplementary Table 3: Gene acronyms for four *BnITPK* genes used in this study. All mutant alleles are listed with their effects on their protein sequence.

Gene	Acronym	Allele	Type of mutation	Mutation position (upstream of PAM site)	Type of change in protein structure*
Bn.ITPK1.A10	*A*	A_h	Wild type (Haydn)	-	no change
		A_1	1bp deletion	3	aa change at 146 and premature stop at 160
		A_2	2bp deletion	3,4	aa change at 146 and premature stop at 148
		A_3	3bp deletion	3	P146T
		A_4	GTTT insertion	from 3rd base	aa change at 146 and premature stop at 150
		A_5	A insertion	3	premature stop codon at 150
		A_6	T insertion	3	premature stop codon at 150
		A_7	10bp deletion	from 3rd base	premature stop codon at 156
Bn.ITPK1.C09	*B*	B_h	Wild type	-	no change
		B_1	C insertion	5	aa change at 146 and premature stop at 150
		B_2	A insertion	4	aa change at 146 and premature stop at 150
		B_3	T insertion	4	aa change at 146 and premature stop at 150
		B_4	2bp deletion	3,4	aa change at 146 and premature stop at 148
		B_5	GTT insertion	5	aa change at 146 and premature stop at 150
		B_6	G insertion	3	aa change at 146 and premature stop at 148
		B_7	6bp deletion	from 3rd base	loss of two aa (KP at 144, 145)
		B_8	1bp deletion	3	aa change at 146 and premature stop at 150
Bn.ITPK4.A05	*E*	E_h	Wild type (Haydn)	-	no change
		E_1	A insertion	3	aa change at 36 and premature stop codon at 138
		E_2	AA insertion	3,4	premature stop codon at 44

		E₃	T insertion	3	aa change at 35 and premature at 149 position
		E₄	T insertion	6	aa change at 35 and premature at 149 position
Bn.ITPK4.C04b	*G*	G_h	Wild type (Haydn)	-	no change
		G_1	AA insertion	3,4	aa change at 31 and premature stop codon at 38
		G_2	T insertion	3	aa change at 31 and premature stop codon at 38
		G_3	TT insertion	3,4	aa change at 31 and premature stop codon at 38
		G_4	T insertion	5	aa change at 31 and premature stop codon at 38
		G_5	4bp deletion	from 4th base	aa change at 29 and premature stop at 36
		G_6	3bp deletion	from 3rd base	loss of one aa (I at 29)
		G_7	6bp deletion	from 4th base	loss of two aa (LI at 28,29)
		G_8	A insertion	4	aa change at 31 and premature stop codon at 38

*aa stands for amino acids and deletion of respective amino acids are shown by single letter amino acid code

Supplementary Table 4: Segregation analysis in T_3 generations

seed code	No. of plants	Transgene genotype		χ^2 (3:1)	*ITPK* genotypes		
		Transgenic	Non-transgenic				
180705	10	8	2	0.13	$A_5A_5B_2B_2E_hE_hG_hG_h$	$A_5A_5B_2B_hE_hE_hG_hG_h$	$A_5A_5B_hB_hE_hE_hG_hG_h$
					2	5	3
180716	10	7	3	0.13	$A_1A_1B_3B_3E_hE_hG_8G_8$	$A_1A_1B_3B_7E_hE_hG_8G_8$	$A_5A_5B_7B_7E_hE_hG_hG_8$
					4	2	4
180663	10	9	1	1.2	$A_hA_7B_hB_3E_hE_hG_hG_2$	$A_hA_5B_hB_3E_hE_hG_hG_h$	$A_hA_6B_hB_3E_hE_hG_hG_2$
					7	1	2

Three populations were genotyped with locus specific primer combinations. The significance of the chi-square test was validated at p=0.05.
